# Study of patients with cleft lip and palate with consanguineous parents

**DOI:** 10.1590/S1808-86942011000100004

**Published:** 2015-10-19

**Authors:** Sibele Nascimento de Aquino, Lívia Máris Ribeiro Paranaíba, Daniella Reis Barbosa Martelli, Mário Sérgio Oliveira Swerts, Letízia Monteiro de Barros, Paulo Rogério Ferreti Bonan, Hercílio Martelli Júnior

**Affiliations:** 1 Graduate student in stomatopathology, Campinas State University (Universidade Estadual de Campinas) - Unicamp; 2 Doctoral student of stomatopathology, Campinas State University - Unicamp; 3 Professor of semiology, Montes Claros State University (Universidade Estadual de Montes Claros) - Unimontes; 4 Professor at the Alfenas University (Universidade de Alfenas) - Unifenas; 5 Professor at the Alfenas University (Universidade de Alfenas) - Unifenas; 6 Professor at the Montes Claros State University - Unimontes; 7 Doctoral degree, full professor, Montes Claros State University - Unimontes

**Keywords:** consanguinity, cleft lip, cleft palate

## Abstract

Cleft lip and/or palate (CL/P) are the most common congenital anomalies of the face. CL/P are non-syndromic (CL/PNS) in about 70% of subjects.

**Aim:** To describe clinical cases of non-syndromic CL/P (CL/PNS) associated with consanguinity, diagnosed at a reference hospital in Minas Gerais, Brazil, and to correlate these alterations with possible risk factors.

**Series and Methods:** A retrospective study at a reference hospital for craniofacial deformities in Minas Gerais, Brazil from 2006 to 2009 based on data in medical records.

**Results:** Of 246 CL/PNS cases diagnosed and treated at the institution, 15 (6.1%) were CL/PNS with reported first-degree consanguinity; 73.3% occurred in males. Four of 15 patients had complete right cleft palate and lip (CLP), 4 presented complete cleft palate and lip (right and left), 3 had complete unilateral left CLP, 3 had isolated cleft palate, and 1 presented cleft lip only. Among the risk factors, only three mothers reported smoking during pregnancy.

**Conclusions:** CLP (unilateral or bilateral) were more frequent in the group with a history of first-degree consanguinity; males predominated. Among the risk factors, only smoking was observed in three cases.

## INTRODUCTION

Non-syndromic cleft lip and palate (NSCLP) - OMIM 119530 - are the most frequent craniofacial anomalies. The occurrence of CLP in several regions of the world is higher than that of Down syndrome.^1^ The incidence of CLP é about 1 in every 500 - 2,000 live births, and varies depending on race, social-economic status and population.^2^, ^3^, ^4^ Epidemiologic studies in Brazil have shown that the incidence of CLP ranges from 0.19 to 1.54 for every 1,000 live births.^5,^^6^ About 70% of CLP cases are nonsyndromic; the other 30% are syndromic clefts.^7^, ^8^, ^9^ Over 300 syndromes (including chromosomal or Mendelian disorders) include CLP in its clinical spectrum.^8,^^10^

CLP derive embryologically from primary fusion defects of the craniofacial processes that form the primary and secondary palate in the first trimester of intrauterine development.^8^ CLP are clinically classified, based on the anatomy of the incisive foramen, into four groups: preincisive foramen or clefts lip (CL), post-incisive foramen or cleft palate (CP), trans-incisive foramen or cleft lip and palate (CLP), and rare facial clefts.^11^

The pathogenesis is poorly understood, as CLP arise depending on multiple genetic and environmental factors. The mother's diet, vitamin supplementation, use of alcohol, smoking, and anticonvulsant drugs are some of the environmental factors that have been related with clefts.^10,^^12^ Genetic factors include several craniofacial formation genes, such as: TGF-b3 (Transforming growth factor beta 3),^13^ MSX1 (Msh homeobox 1),^14^ IRF6 (Interferon regulatory factor 6),^15^ FGFs (Fibroblast growth factor),^16^ PVRL1 (Poliovirus receptor related-1),^10^ FOXE1 (Forkhead box E1), JAG2 (Jagged 2)^17^ and TBX22 (T-box 22).^18^

In addition to these environmental and genetic factors there is the association between CLP and consanguinity.^19^, ^20^, ^21^ Consanguinity is considered a significant factor in autosomal recessive diseases; it has also been associated with congenital anomalies such as hydrocephalus, polydactilia and CLP.^19,^^22^ The risk of congenital conditions is higher in subjects born of first degree consanguineous parents compared with those of non-consanguineous marriages.^22^

The purpose of this study was to describe a clinical case series of NSCLP with a history of first degree consanguinity, at a Brazilian referral unit for craniofacial deformities.

## SERIES AND METHODS

A retrospective study was made of cases seen at a referral center for craniofacial anomalies from 2006 to 2009 in the state of Minas Gerais, Brazil. There were 246 NSCLP cases in that period, of which 15 patients (6.1%) had a history of first degree consanguinity between parents. Patients with syndromic CLP and those with other uncommon additional features to clefts were excluded.

A questionnaire with the variables age, sex, skin color, history of consanguinity, type of NSCLP, age of mother and father during gestation, smoking and alcohol use by mother during pregnancy to assess the risk factors for NSCLP and to gather data. Based on the incisive foramen as an anatomical landmark, clefts were classified into:^11^ (1) CL: include unilateral or bilateral complete or incomplete pre-foramen clefts; (2) CLP: include unilateral or bilateral trans-foramen and pre- and post-foramen clefts; (3) CP: include all complete or incomplete post-foramen clefts, and (4) other, which were rare facial clefts.

After applying the questionnaires, data were archived in a database and analyzed using the SPSS^®^ version 17.0 (Chicago, EUA) statistical software package. The institutional review board of the university approved this study (# 26/2005).

## RESULTS

From 2006 to 2009, 246 NSCLP patients visited the Craniofacial Anomaly Unit. Of these, 15 (6.1%) patients had clefts and a history of first degree consanguinity among parents. Table 1 presents the sex, skin color and age data of patients; 11 were male (73.3%) and 4 were female (26.7%). There were 10 patients (66.7%) of brown skin color, 4 white patients (21.4%) and 1 black patient (6.7%). Most patients (n=7) were aged from 11 to 20 years.

**Table 1:** General features of 15 clinical of non-syndromic cleft lip and palate cases

		n	%
Sex	Male	11	73,3
	Female	4	26,7
Age range (years)	≤ 10	4	26,7
	>11≤20	7	46,7
	>21≤30	3	20
	>31	1	6,7
Skin color	White	4	26,7
	Brown	10	66,7
	Black	1	6,7
	CP (isolated) incomplete	3	20
Type of cleft	CL (isolated) unilateral complete	1	6,7
	CLP left unilateral complete	3	20
	CLP right unilateral complete	4	26,7
	CLP bilateral complete	4	26,7
Total		15	100

Table 1 shows the prevalence of NSCLP in the sample and the percentages of types of cleft. CLP was more frequent compared to isolated CP and CL; 4 patients (26.7%) had unilateral right complete CLP, 4 patients (26.7%) had bilateral complete CLP, 3 patients (20%) have unilateral left complete CLP, 3 patients (20%) had isolated CP, and 1 patient (6.7%) had isolated CL.

Table 2 presents the distribution of parent's ages during pregnancy. The numbers in mother's age groups 21 to 25 years, 26 to 30 years, and 31 to 35 years were similar. The most common age ranges for fathers were 26 to 30 years and 31 to 35 years.

**Table 2:** Distribution of paternal and maternal ages during pregnancy

		n	%
Maternal age (years)	≤20	2	13,3
	21 a 25	5	33,3
	26 a 30	4	26,7
	31 a 35	4	26,7
Paternal age (years)	21 a 25	3	20
	26 a 30	4	26,7
	31 a 35	4	26,7
	36 a 40	3	20
	41 a 45	1	6,7
Total		15	100

There was no history of alcohol or drug use by mothers during pregnancy. Three mothers (20%) reported smoking during pregnancy, of which 2 mothers (13.3%) smoked more than five cigarettes a day. All patients and relatives were attended and are monitored at a multiprofessional clinical team.

## DISCUSSION

Recent studies have suggested that many genes and certain environmental factors are involved in the etiology of NSCLP.^8,^^10,^^23^ Estimates have suggested that 3 to 14 genes may be involved in CLP.^4,^^24^ Besides genes and environment, consanguinity has also been implicated in CLP and other congenital anomalies.^2,^^20,^^25^ The risk of congenital disorders is about 2 to 3% in non-consanguineous marriages, and 5 to 8% in first degree consanguineous marriages.^22^

**Table 3:** Smoking in pregnant subjects

		n	%
Smoking during pregnancy	Yes	3	20
	No	12	80
	≤ 5 cigarettes per day	1	6,7
Quantity of cigarettes	> 6 cigarettes per day	2	13,3
	Not applies	12	80
Total		15	100

There were 246 cases of cleft patients seen at out unit from 2006 to 2009; 15 of which (6.1%) had a history of consanguinity. Epidemiologic and genetic studies of 207 NSCLP cases in France revealed that 3.9% of patients had a history of first degree consanguinity among parents; a positive association was found between consanguinity and NSCLP. These authors suggested that this association is probably related with a recessive genetic component and environmental factors.^26^ A study in Teheran included 25 NSCLP cases in an 8-year period, of which 31.8% were from consanguineous marriages. Among the group of children without NSCLP, only 8% came from consanguineous marriages.^25^

The relationship between congenital anomalies and consanguinity has been noted in other papers. A study on the presence of congenital anomalies in children of consanguineous marriages found a significant association between first degree consanguinity and anomalies such as cerebral palsy, cystic fibrosis, physical retardation, congenital blindness, and NSCLP.^22^ A Latin American study found that 5,931 children (of 32,845 children) came from consanguineous marriages and had congenital anomalies. NSCLP were reported - among others - as congenital anomalies associated with consanguinity.^19^

The types of clefts are distributed differently; the incidences vary in different population groups.^24,^^27^, ^28^, ^29^ Our findings showed that there were more CLP compared with isolated CP or CL; this result is similar to that of Souza-Freitas et al.^30^ On the other hand, Stoll et al.^26^ found higher rates of isolated CP compared with CLP. The extension and location were as follows: 3 patients (20%) with unilateral left complete CLP, 4 patients (26.7%) with unilateral right complete CLP, 4 patients (26.7%) with bilateral complete CLP, 3 patients (20.05%) with isolated CP, and 1 patient (6.7%) with isolated CL. Stoll^26^ and Jamilian et al.^25^ found lower rates for CL, as was the case in our sample. Some studies have suggested that unilateral CLP (left or right) are more frequent that bilateral CLP.^26,^^31^

Eleven of our 15-patient samples were male and 4 were female. A few studies^26,^^31^ have shown that the prevalence of this condition in males is generally higher. Our sample had more male subjects in all types of cleft. Some studies^4,^^30,^^32^ have shown that isolated CP occurs more typically in females, and CLP in males. A study of 126 Brazilian children with NSCLP found that the prevalence of CLP in males was 2.57 times that of females.^31^

A higher age of the mother is considered a risk factor for several chromosomal and non-chromosomal abnormalities; there is, however, no consensus as to whether maternal age is a risk factor for CLP.^33^ In our sample only one father was aged 41 to 45 years (Table 2). A study of 100 children with CLP revealed that maternal age was significant for the occurrence of clefts, and that ages 26 to 35 years and over 35 years had a lower risk for CLP compared to women aged up to 25 years.^12^ On the other hand, a study by Vieira et al.^34^ found no general association between maternal age and CLP.

A few environmental risks have been related with NSCLP, such as maternal diet, vitamin supplementation during pregnancy, use of alcohol, anticonvulsant drugs, and smoking.^10,^^27,^^35^ Our findings showed that no mother consumed alcoholic beverages or took anticonvulsants during pregnancy. Three mothers (20%) reported smoking during the entire pregnancy; two (13.3%) smoked more than 5 cigarettes a day. Maternal smoking during pregnancy has been associated with an increased risk of CLP.^9,^^21,^^28^ Considering the multifactor etiology of NSCLP, this study evaluated the presence of consanguinity and risk factors in clinical cases of NSCLP, showing that notwithstanding the limited number of cases (n=15), it is important to describe these clinical cases to further our understanding of consanguinity associated with NSCLP.

## CONCLUSION

This study analyzed 15 clinical cases of NSCLP with a history of first degree consanguinity among parents. Unilateral or bilateral CLP were the most frequent in this group, followed by isolated CP. Males predominated in all types of clefts. As risk factors, only smoking during pregnancy was reported by three mothers. Further studies may shed additional light on the relationship between consanguinity and NSCLP.

## ACKOWLEDGEMENTS

To the Fundação de Amparo à Pesquisa do Estado de Minas Gerais (Fapemig) and the Conselho Nacional de Desenvolvimento Científico e Tecnológico (CNPq) (HMJ).
